# Dielectric Spectroscopy of Water Dynamics in Functionalized UiO-66 Metal-Organic Frameworks

**DOI:** 10.3390/molecules25081962

**Published:** 2020-04-23

**Authors:** Sergejus Balčiūnas, Diana Pavlovaitė, Martynas Kinka, Jyun-Yi Yeh, Po-Chun Han, Fa-Kuen Shieh, Kevin C.-W. Wu, Mantas Šimėnas, Robertas Grigalaitis, Jūras Banys

**Affiliations:** 1 Faculty of Physics, Vilnius University, Sauletekio av. 9, LT-10222 Vilnius, Lithuania; sergejus.balciunas@ff.vu.lt (S.B.); diana.pavlovaite@ff.stud.vu.lt (D.P.); martynas.kinka@ff.vu.lt (M.K.); mantas.simenas@ff.vu.lt (M.Š.); robertas.grigalaitis@ff.vu.lt (R.G.); 2 Department of Chemical Engineering, National Taiwan University, Taipei 10617, Taiwan; d07551002@ntu.edu.tw (J.-Y.Y.); a242520002000@gmail.com (P.-C.H.); kevinwu@ntu.edu.tw (K.C.-W.W.); 3 Department of Chemistry, National Central University, Chung-Li 32001, Taiwan; fshieh@cc.ncu.edu.tw

**Keywords:** metal-organic framework, UiO-66, dielectric spectroscopy, water dynamics

## Abstract

We present a dielectric spectroscopy study of dipolar dynamics in the hydrated UiO-66(Zr) type metal-organic frameworks (MOFs) functionalized with −NH_2_ and −F groups. Experiments are performed in a broad temperature and frequency ranges allowing us to probe several dipolar relaxations. For both samples at temperature below 220 K, we observe confined supercooled water dynamics, which can be described by the Arrhenius law. At slightly higher temperature, a second less pronounced dipolar relaxation is identified, and its origin is discussed. At even higher temperature, the dielectric permittivity exhibits anomalous increase with increasing temperature due to the proton conductivity. Upon further heating, the permittivity shows a sudden decrease indicating a reversible removal of water molecules. Measurements of the dehydrated samples reveal absence of all three dipolar processes.

## 1. Introduction

Metal-organic frameworks (MOFs) are crystalline compounds consisting of metal centers joined together by organic linkers into highly porous structures [1]. A vast diversity of these building units allows synthesis of various MOF structures with different topologies and functionalities [2]. The high porosity and tunability of these compounds is expected to be utilized for selective gas adsorption [3,4], separation [5], and storage [6,7] as well as chemical catalysis [8], drug delivery [9] and other applications [10].

**Figure 1:**
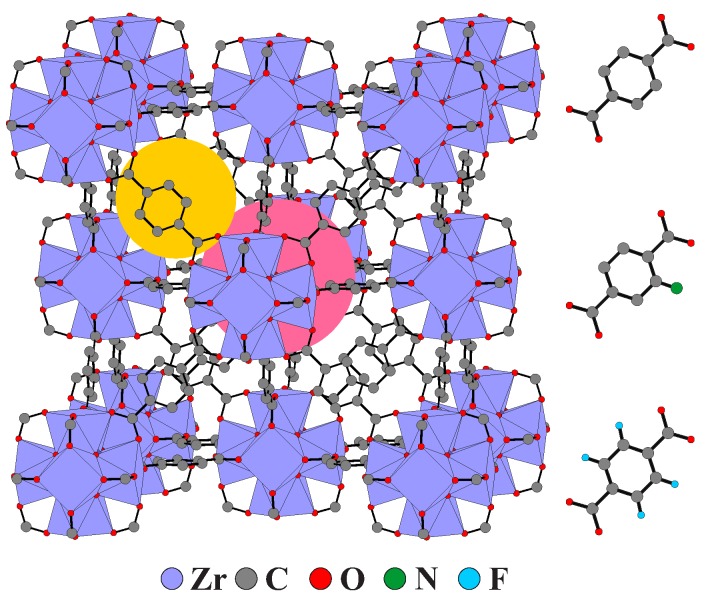
Crystal structure of UiO-66 (left). Examples of octahedral and tetrahedral pores are indicated in pink and yellow, respectively. BDC linker and its functionalized BDC−NH_2_ and BDC−F_4_ analogues (right). Structural data taken from Ref. [11]. Hydrogen atoms of BDC linkers are not shown for clarity.

A highly promising family of such compounds is UiO MOFs (UiO stands for University of Oslo), which exhibit superior thermal and chemical stability as well as high porosity [11]. One of the most popular members of these compounds is UiO-66(Zr) MOF consisting of Zr_6_O_4_(OH)_4_ secondary building units (SBUs), which are connected by 1,4-benzene-dicarboxylate (BDC) linkers into porous structures with $Fm\bar{3}m$ face-centered cubic structure (see Figure 1) [11]. The framework has octahedral pores connected to smaller tetrahedral cavities via relatively narrow windows of about 5 Å diameter. The pronounced stability of UiO-66 stems from the robust SBUs and their high coordination number, which results in the exceptional defect tolerance [12,13].

The gas adsorption, gas separation as well as other physical and chemical properties of UiO-66 MOF can be significantly modified by introducing functionalized BDC linkers with various chemical groups (e.g., −NH_2_, −OH, −Br, −COOH, −NO_2_ and −F), while retaining the stability of the framework [14,15,16,17]. For example, functionalization by −NH_2_ group results in a 20-fold increase in phosphate-ester hydrolysis rate [18], increased capacity to detect explosive simulants [19], better mechanical stability [20] and significant improvement of CO_2_/CH_4_ separation performance [21,22] compared with pristine UiO-66 MOF.

Attachment of the functional groups is also expected to influences the linker dynamics in UiO MOFs. In addition to NMR spectroscopy [23,24] and quasi-elastic neutron scattering [25], a method of choice to study such dynamic effects of polar linkers and guest molecules is dielectric spectroscopy [26,27,28,29]. This technique was used to probe the linker dynamics of functionalized UiO-66 with polar −Br, −OH and −NH_2_ groups [30]. In addition, the same method was also employed to study dynamics of the biologically active guest molecules such ibuprofen and caffeine adsorbed in UiO-66-NH_2_ MOF [31,32]. The dielectric spectroscopy also revealed water dynamics and enhancement of proton conductivity in UiO-66 with acidic −COOH polar groups [33]. Two dipolar relaxation processes on different time scales were observed and assigned to dynamics of the confined water clusters and cooperative motion of the water molecules bound to the pore wall.

Despite these investigations, the dynamic and sorption properties of water in other functionalized analogues of UiO-66 are poorly studied, though elucidation of such processes is essential for applications of these and other MOFs for CO_2_ adsorption and separation [4]. Thus, in this work, we use dielectric spectroscopy to probe the dipolar dynamics in the hydrated functionalized UiO-66-NH_2_ and UiO-66-F_4_ MOFs in a broad temperature and frequency ranges. Both functional groups are expected to exhibit different degree of hydrophobicity motivating their choice for this study. We reveal three different dynamic processes of water, which are related to the supercooled confined water and proton conductivity.

## 2. Results and Discussion

First we performed temperature dependent dielectric spectroscopy experiments of UiO-66-NH_2_ and UiO-66-F_4_ powder samples starting from the hydrated forms. Before the measurements, samples were kept in a high humidity atmosphere for 24 h to achieve maximum hydration level. The measurement cycle started by cooling from the room temperature to about 125 K at ambient atmosphere followed by heating to above 400 K.

**Figure 2:**
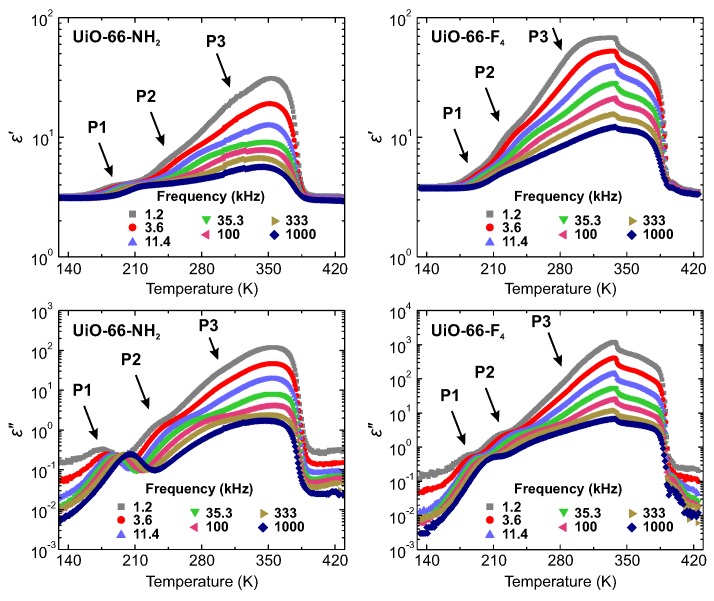
Temperature dependence of the complex dielectric permittivity of UiO-66-NH_2_ and UiO-66-F_4_ hydrated powder samples at several selected frequencies. Three processes, P1, P2 and P3, related to the adsorbed water are indicated by the arrows. Measurements performed on heating.

The temperature dependences of the real $\varepsilon^{\prime }$ and imaginary $\varepsilon^{\prime \prime }$ parts of the complex dielectric permittivity $\varepsilon^{*}=\varepsilon^{\prime }-i\varepsilon^{\prime \prime }$ of hydrated UiO-66-NH_2_ and UiO-66-F_4_ samples on heating are presented in Figure 2. Both samples reveal three frequency dependent processes. The weakest dispersive process P1 is visible below 220 K. With increasing temperature, the second process P2 enters our measurement frequency window in the 220–250 K temperature region and is superimposed by the strong response of the third process P3 at higher temperature.

**Figure 3:**
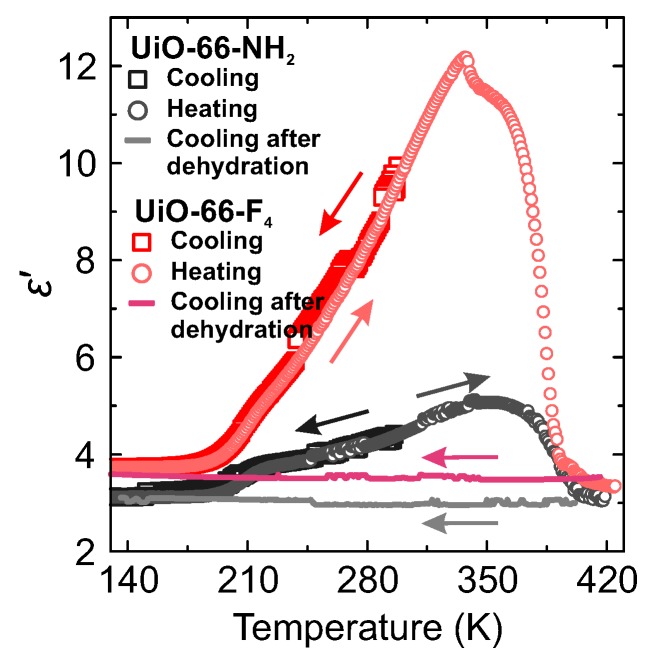
Real part of the complex dielectric permittivity of hydrated MOF samples measured at 1 MHz frequency during the cooling-heating-cooling cycle.

The real and imaginary parts of $\varepsilon^{*}$ start to decrease at about 350 K indicating dehydration of the samples [34]. This result is supported by the measurements of the temperature dependent weight loss of hydrated samples (Figure S3), where a significant dehydration continues up to about 370 K. At slightly higher temperature the dielectric permittivity exhibits a sudden drop. A much smaller decrease of $\varepsilon^{*}$ is also observed at about 330 K for UiO-66-F_4_ sample, though its origin is unclear. Subsequently, after the dehydration nitrogen gas was applied and another cooling was performed under nitrogen atmosphere. The obtained flat responses on cooling (Figure 3) indicate that all three processes originate from the adsorbed water molecules within the pores or on the crystallite surfaces. Application of N_2_ gas during the measurements effectively prevents sample rehydration, which usually takes place in MOFs if cooled under ambient air conditions [28,35].

**Figure 4:**
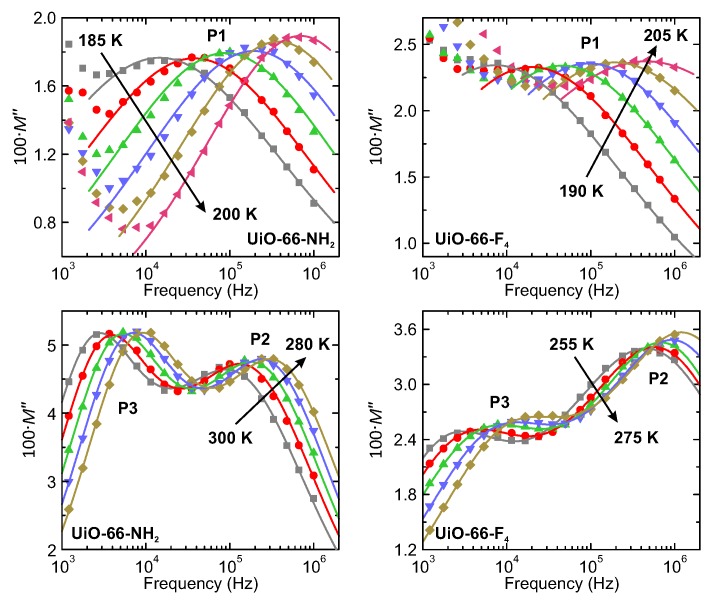
Frequency dependence of the imaginary part of the complex electric modulus of UiO-66-NH_2_ and UiO-66-F_4_ hydrated MOFs presented at selected temperatures. Relaxation processes are indicated by the labels. Solid lines the best fits to the Cole-Cole relaxation processes.

The three processes observed in the hydrated MOFs were further studied by analyzing the temperature dependences of the dielectric data at different frequencies. The frequency dependence of $\varepsilon^{*}$ is presented in Figures S4 and S5 revealing lack of clearly distinguishable relaxations. Since the dielectric response in our case is likely related to proton transport, we have used the electric modulus $M^{*}$ representation [36,37]. Generally, for a dielectric relaxation process, a relaxation peak appears in both $M^{*}$ and $\varepsilon^{*}$ representations, while for a pure conduction process the peak would be only seen in the $M^{*}$ spectrum. It was demonstrated that the absence of the peak in the imaginary part of $\varepsilon^{*}$ versus frequency shows a long-range conductivity phenomenon, while its presence reveals localized dynamics [38]. In our case, $M^{*}$ representation provides a much better separation of overlapping processes and allows to calculate the main relaxation parameters. The complex electric modulus $M^{*}$ is defined as the inverse of the complex dielectric permittivity $\varepsilon^{*}$:

(1) $$M^{*}=\frac{1}{\varepsilon^{*}}=\frac{\varepsilon^{\prime }}{{\varepsilon^{\prime }}^{2}+{\varepsilon^{\prime \prime }}^{2}}+i\frac{\varepsilon^{\prime \prime }}{{\varepsilon^{\prime }}^{2}+{\varepsilon^{\prime \prime }}^{2}}=M^{\prime }+iM^{\prime \prime },$$

where $M^{\prime }$ and $M^{\prime \prime }$ denote the real and imaginary parts of $M^{*}$, respectively. In our case, all three processes appeared as separated peaks in the frequency dependence of $M^{\prime \prime }$ (Figure 4) demonstrating a clear relaxation character.

The relaxation parameters of each process were extracted by fitting the obtained frequency dependences of $M^{\prime }$ and $M^{\prime \prime }$, where $\varepsilon^{*}$ in Equation (1) was expressed by a superposition of three independent Cole–Cole relaxations [36]:

(2) $$\varepsilon^{*}\left(\omega \right)=\varepsilon \left(\infty \right)+\sum_{n}\frac{\Delta \varepsilon_{n}}{1+{\left(i\omega \tau_{n}\right)}^{1-\alpha_{n}}}.$$

Here, ω is the angular probing frequency, and ε(∞) is the dielectric permittivity in the infinite-frequency limit. The relaxations are described by different dielectric strengths $\Delta \varepsilon_{n}$ and mean relaxation times $\tau_{n}$ (n=1,2,3). The parameters $0<\alpha_{n}\leq 1$ determine the widths of the relaxations.

**Figure 5:**
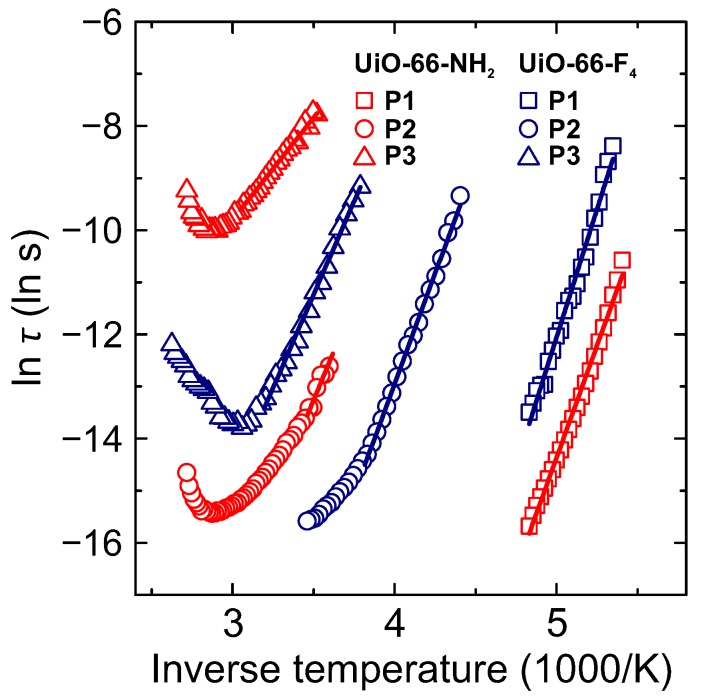
Inverse temperature dependence of the mean relaxation time of P1-P3 processes in hydrated UiO-66-NH_2_ and UiO-66-F_4_ MOFs. The lines are linear fits to the Arrhenius equation.

The obtained temperature dependences of the mean relaxation time for different processes are presented in Figure 5. The low temperature process P1 in both MOFs exhibits the Arrhenius-type temperature dependence of the mean relaxation time: $\tau =\tau_{0}\exp\left(E_{a}/kT\right)$, where $E_{a}$ and $\tau_{0}$ denote activation energy and attempt time, respectively, and *k* is the Boltzmann constant. The determined activation energies for this process are 0.76 and 0.85 eV for UiO-66-NH_2_ and UiO-66-F_4_, respectively (Table 1). Similar values of the activation energies were also reported for the same process in the unfunctionalized UiO-66 and functionalized UiO-66-COOH [33]. For both compounds, the relaxation strength Δε≈1.5 and exponent α≈0.5, which indicates a much broader dispersion than that of the Debye type. The broadening is likely caused by a superposition of more than one relaxation process with different characteristic relaxation times.

A similar response is widely observed for confined supercooled water in disordered porous materials such as silica hydrogels, Vycor glasses, molecular sieves, mineral clay, graphite oxide, cement-like materials and MOFs [28,39,40]. The majority of these materials are hydrophilic, generally because of the hydroxy groups on their surface and possess interconnected pore structures with a broad pore size distribution. In such systems, incomplete water filling of the pores is common and therefore water–surface interactions are promoted causing system-dependent alterations in the dynamical behavior of the confined water [39]. BDC linkers of the non-functionalized UiO-66 are hydrophobic, but the H-bond formation of the confined water molecules with hydroxy groups located at the inorganic nodes may be possible, similar to reported n-butane binding in these MOFs [41] and water molecules in the MIL-53 [42]. The functionalization of the BDC linker with polar F and NH_2_ groups introduces additional possible H-bonding sites in the framework resulting in the increased water–surface interactions. The determined activation energies for both compounds indicate that the dynamics of the confined water molecules are strongly hindered with considerably slower relaxation in UiO-66-F_4_ than in UiO-66-NH_2_ MOF.

A different temperature behavior is obtained for the mean relaxation time of P2 and P3 processes (Figure 5). Starting from the lower temperature, the mean relaxation time follows the Arrhenius law with increasing temperature. However, as temperature approaches the sample dehydration region, a clear minimum of lnτ is observed indicating slowing down of the relaxation upon increase of temperature. High values of $\varepsilon^{\prime }$ at room temperature are commonly observed in various porous materials containing water [26,28,43,44] and are usually interpreted by proton conductivity and/or Maxwell-Wagner polarization. Recent simulations of the proton transfer through the water clusters confined inside a nanometre sized cavities revealed that it proceeds via the Grotthuss diffusion and can even be enhanced, if the confinement is soft, allowing facile hydrogen-bond reorganization [45].

The obtained relaxation parameters of P3 process are strongly influenced by the amount of adsorbed water and different functional groups on the BDC linkers. The determined relaxation times for P3 process can be directly related to the DC conductivity for the charge transfer/relaxation process $\sigma_{DC}∼\tau^{-1}$. Thus, the decrease of the relaxation time with temperature corresponds to the increase fo the DC conductivity with the same activation energy $E_{a}$: $\sigma T∼\exp(-E_{a}/kT)$. The obtained $E_{a}$ value of 0.34 eV for P3 process in hydrated UiO-66-NH_2_ is characteristic for Grotthuss conduction mechanism (typically $E_{a}<0.4$ eV) [46,47].

In general, ionic conductivity can be expressed as σ=Zenμ, where *Z* is the ionic charge, *e* is the elementary charge, *n* denotes the charge carrier concentration and μ is the carrier mobility. Therefore, functionalization of the UiO-66 framework can control proton conductivity by affecting the charge carrier concentration and/or mobility with improved conduction pathways at constant hydration levels. Relatively complicated structure of UiO-66 with interconnected octahedral and smaller tetrahedral pores may result in different intra-cage, inter-cage or window crossing mechanisms of proton transfer, similar as for a range of crystalline porous organic cages at high hydration levels [45]. High $E_{a}$ value for P3 process in UiO-66-F_4_ suggests that proton mobility could be also related to the pore and window size, which decreases after functionalization hindering the long range proton transfer. At temperature higher than 330 K, where water desorption process starts dominating, water loss gradually breaks proton transfer pathways, which results in the observed slowing down of the relaxation time and lowering of the overall conductivity.

Interestingly, process P2 in both systems also displays qualitatively similar response to that of the process P3. Note that at lower temperatures, where process P2 enters our measurement frequency window, observation of linker rotation dynamics in functionalized UiO-66 MOFs was reported [30]. However, our dielectric measurements of the dehydrated samples (Figure 3) do not show any relaxation process due to the frequency dependent linker motion (at least in the sensitivity range of our measurement setup). In addition, process P2 in UiO-66-COOH MOF was ascribed to the dynamics of water/solid surface complex [33]. Similarity between the temperature dependences of the processes P2 and P3 indeed demonstrate that the origin of P2 process can be related to water conductivity response. In Grotthuss mechanism, the proton transfer through H-bonding network may be accompanied by a simultaneous sequential rotations of the molecules [48]. Ligand functionalization by polar groups can result not only in the creation of the additional charge carriers, but also in appearance of additional binding sites for possible H-bond formation with adsorbed water molecules and development of new enhanced proton transfer routes.

**Figure 6:**
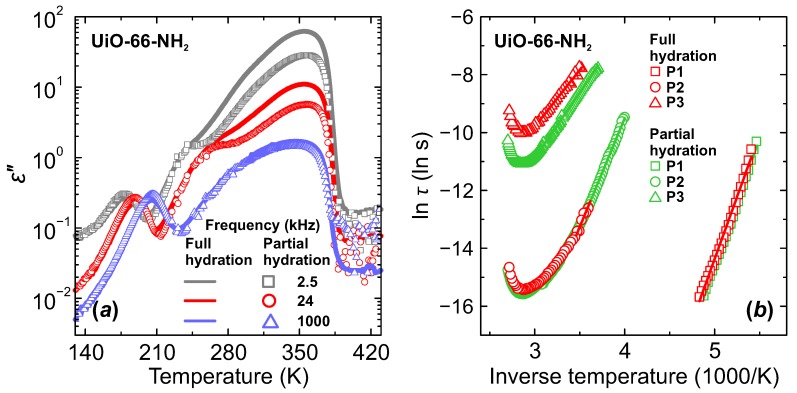
Temperature dependence of the (**a**) imaginary part of the complex dielectric permittivity and (**b**) mean relaxation time of fully and partially hydrated UiO-66-NH_2_ MOF.

In order to further elucidate the corresponding relaxation mechanisms, we performed dielectric measurements on UiO-66-NH_2_ and UiO-66-F_4_ MOF samples with different adsorbed water content. After activation at about 420 K, samples were cooled down to room temperature and left in the ambient atmosphere for 24 h to let natural water uptake. Afterwards, the same cooling-heating measurement cycle was performed. The obtained temperature and frequency dependences of the complex dielectric permittivity were compared with the ones recorded for highly hydrated samples. As can be seen from Figure 6a, only the low frequency process P3 was altered by insufficient hydration. Sample treatment in lower humidity atmosphere results in lower eps values, which is a clear indication of lower hydration level as reported for other porous compounds [26,28,43,44].

For P1 and P2 processes we observed no significant influence of the hydration level on their dielectric response or relaxation parameters, except that we were able to trace the P2 process to lower temperature due to less pronounced P3 process (Figure 6). These observations further imply that P1 and P2 processes could be related to water molecules near the pore surface. Our dielectric results suggest that at first the interfacial layers or sites are filled before formation of the proton transfer chain networks at higher hydration levels. Molecular dynamics simulation of microscopic mechanisms associated with the water-mediated proton transport in the MIL-53 MOF also show that at low water content water molecules tend to concentrate near the proton binding sites of the framework [42]. The observation that P2 has the same relaxation rate at different hydration levels (Figure 6b) suggests that this process can be related to water molecules that are situated close to the pore surface binding sites and form water/ligand H-bonded network for proton transfer. This process appears much earlier in the measured dielectric spectra with increasing temperature than P3 process, but has much higher activation energy (Table 1) in both UiO-66-NH_2_ and UiO-66-F_4_ MOFs, confirming stronger water–framework interaction influence.

When assessing the impact of linker modification on the dielectric permittivity data of adsorbed water, we must also stress that although −F group is considered much more hydrophobic, it still participates in the observed conduction and relaxation processes of confined water. Recent studies show that under certain circumstances, fluorine can act as a hydrogen bond acceptor [49]. Our results suggest that both −NH_2_ and −F groups influence the dynamics of the confined water molecules in qualitatively the same way by introducing additional binding sites for possible H-bond formation compared to unmodified UiO-66 MOF. The relatively weaker H-bond acceptor performance of −F results in slower relaxation of supercooled water molecules (process P1) and much higher activation energy for P3 proton transfer process in UiO-66-F_4_, compared with UiO-66-NH_2_, where all three water–MOF interactions are further enhanced because of a larger hydrophilicity of this group.

## 3. Materials and Methods

### 3.1. Sample Preparation

UiO-66-NH_2_: zirconyl chloride octahydrate (21 mg) was dissolved in DMF (3 mL) at 50–60 ${}^{\circ }$C. This solution was added to a DMF solution (1 mL) in which 2-aminoterephthalic acid (54 mg) was dissolved completely. The combined mixture was homogenized by swirling before a small amount of glacial acetic acid (0.98 mL) was added. The resulting mixture was sonicated at 50–60 ${}^{\circ }$C and placed in an oil bath (120 ${}^{\circ }$C) under static condition for 24 h. After cooling to room temperature, the precipitate was collected by centrifugation and washed with methanol three times. The isolated solid was then dried at room temperature under vacuum to give a yellow powder.

UiO-66-F_4_: tetrafluoroterephthalic acid (30 mg) and zirconyl nitrate hydrate (45 mg) were separately added into deionized water (1.0 mL for each). After mixing these two solutions, a small amount of glacial acetic acid (0.25 mL) was added into the mixture. After stirring for 40 h at room temperature, the precipitate was collected by centrifugation. The as-synthesized sample was washed with D.I. water 3 times and then sequentially washed with ethanol 2 times, and finally immersed in acetone for 2 days. The isolated solid was then dried at room temperature under vacuum to give a white powder.

The scanning electron microscope (SEM) images (Figure S1 in the Supporting Information) revealed uniform size and shape of the crystallites. The average particle diameter is about 100 and 500 nm for UiO-66-NH_2_ and UiO-66-F_4_ samples, respectively. The obtained powder X-ray diffraction (PXRD) patterns of both synthesized compounds are typical for UiO-66 (see Figure S2).

### 3.2. Dielectric Spectroscopy

Dielectric spectroscopy measurements of UiO-66-NH_2_ and UiO-66-F_4_ powders were performed in 1 kHz–1 MHz frequency and 130–430 K temperature ranges using a custom made cryostat and HP 4284A precision LCR meter. The sample was placed and slightly pressed using about 90 kPa pressure in a cylindrical powder holder with top and bottom circular brass electrodes of 6 mm diameter. The typical sample thickness was 1.5 mm. All experiments were performed at 1 K/min cooling and heating rate. Measurements of the hydrated samples above room temperature were carried out in air, while a constant flow of nitrogen gas was used below this temperature and during the experiments with the dehydrated MOFs.

## 4. Conclusions

In summary, we investigated the dielectric response of functionalized UiO-66-NH_2_ and UiO-66-F_4_ MOFs. After hydration, both compounds exhibit a huge dielectric dispersion which can be separated into three partially overlapping processes. Sample dehydration causes disappearance of all these processes demonstrating that their origin is adsorbed water molecules. A detailed analysis of the mean relaxation times obtained from the frequency dependent data revealed that linker functionalization plays a significant role in affecting confined water cluster relaxation (P1 process) as well as the overall proton migration (processes P2 and P3). It is also plausible that linker functionalization can create new pathways for proton migration and potentially enhance the electrical conductivity. This observation is highly important for further application of UiO and similar funtionalized MOFs in electronic devices and sensors.

## Figures and Tables

**Table 1:** Attempt time and activation energy for different processes in hydrated UiO-66-NH_2_ and UiO-66-F_4_ MOFs.

UiO-66-NH_2_		
Process	$\tau_{0}$ (s)	$E_{a}$ (eV)
P1	$1.1\left(1\right)\times 10^{-25}$	0.76(2)
P2	$6.7\left(1\right)\times 10^{-18}$	0.65(1)
P3	$4.4\left(1\right)\times 10^{-10}$	0.34(1)
**UiO-66-F_4_**		
Process	$\tau_{0}$ (s)	$E_{a}$ (eV)
P1	$2.6\left(1\right)\times 10^{-27}$	0.85(2)
P2	$4.6\left(1\right)\times 10^{-21}$	0.73(1)
P3	$1.7\left(1\right)\times 10^{-16}$	0.62(1)

## Supplementary Materials

The following are available online, Figure S1: SEM images of UiO-66-NH_2_ and UiO-66-F_4_ MOF crystallites, Figure S2: PXRD patterns of UiO-66-NH_2_ and UiO-66-F_4_ MOFs, Figure S3: Temperature dependence of the weight loss of hydrated UiO-66-NH_2_ and UiO-66-F_4_ MOFs, Figure S4: Frequency dependence of the complex dielectric permittivity of UiO-66-NH_2_ hydrated MOF, Figure S5: Frequency dependence of the complex dielectric permittivity of UiO-66-F_4_ hydrated MOF.

## Abbreviations

The following abbreviations are used in this manuscript:

MOF	Metal-organic framework
SBU	Secondary building unit
BDC	1,4-benzene-dicarboxylate
