# Supplementary material for: Dielectric Spectroscopy of Water Dynamics in Functionalized UiO-66 Metal-Organic Frameworks
Source: Molecules. 2020 Apr 23;25(8):1962. doi: 10.3390/molecules25081962 (PMC7221552; doi:10.3390/molecules25081962)
Supplement: Supplementary file 1 [file molecules-25-01962-s001.pdf]

Supplementary information:
Dielectric spectroscopy of water dynamics in functionalized UiO-66 metal-organic frameworks

Sergejus Balčiūnas, Diana Pavlovaitė, Martynas Kinka, Jyun-Yi Yeh, Po-Chun Han, Fa-Kuen Shieh, Kevin C.-W. Wu, Mantas Šimėnas, Jūras Banys*, Robertas Grigalaitis

*juras.banys@ff.vu.lt

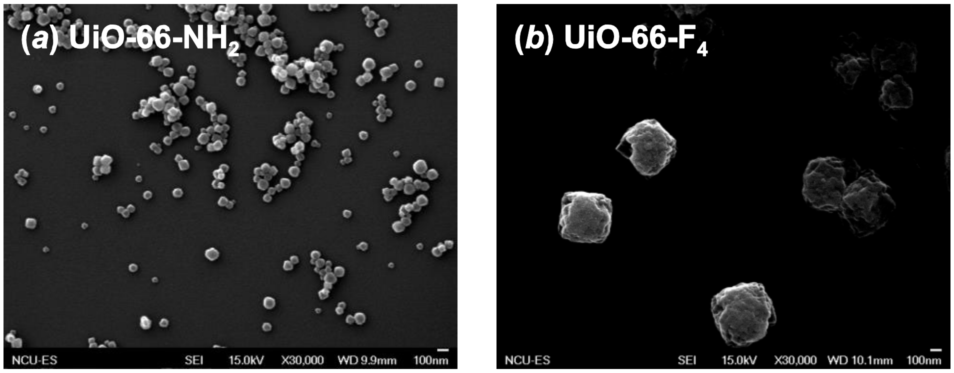

Figure S1. SEM images of (*a*) UiO-66-NH$_2$ and (*b*) UiO-66-F$_4$ MOF crystallites. Experiments were performed using NovaTM NanoSEM.

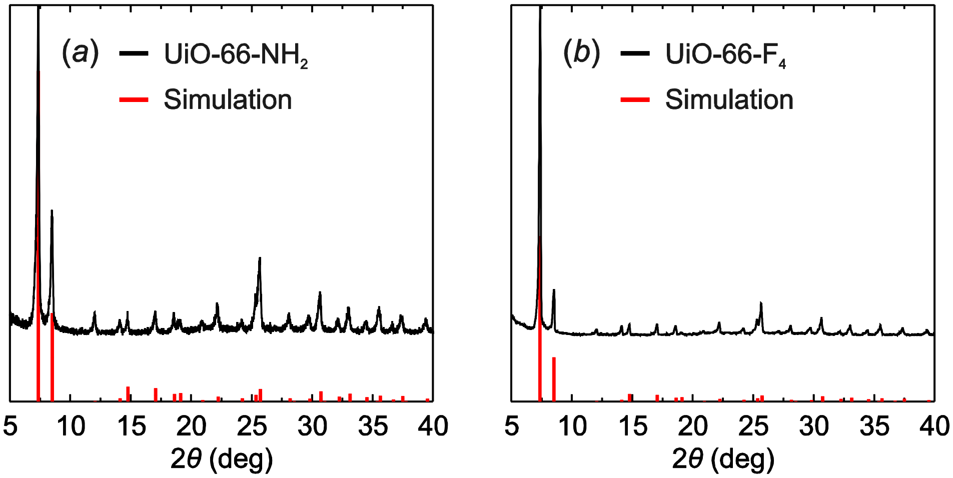

Figure S2. PXRD patterns of (*a*) UiO-66-NH$_2$ and (*b*) UiO-66-F$_4$ MOFs. Measurements performed using Cu K-α diffractometer.

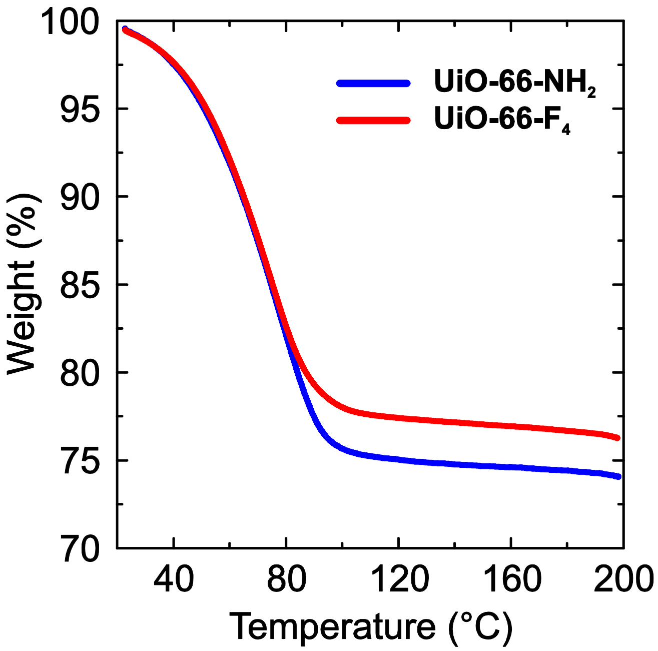

Figure S3. Temperature dependence of the weight loss of hydrated UiO-66-NH_2 and UiO-66-F_4 MOFs. Measurements performed using Perkin Elmer STA6000 thermal analyzer. Measured mass of each sample was 5 mg.

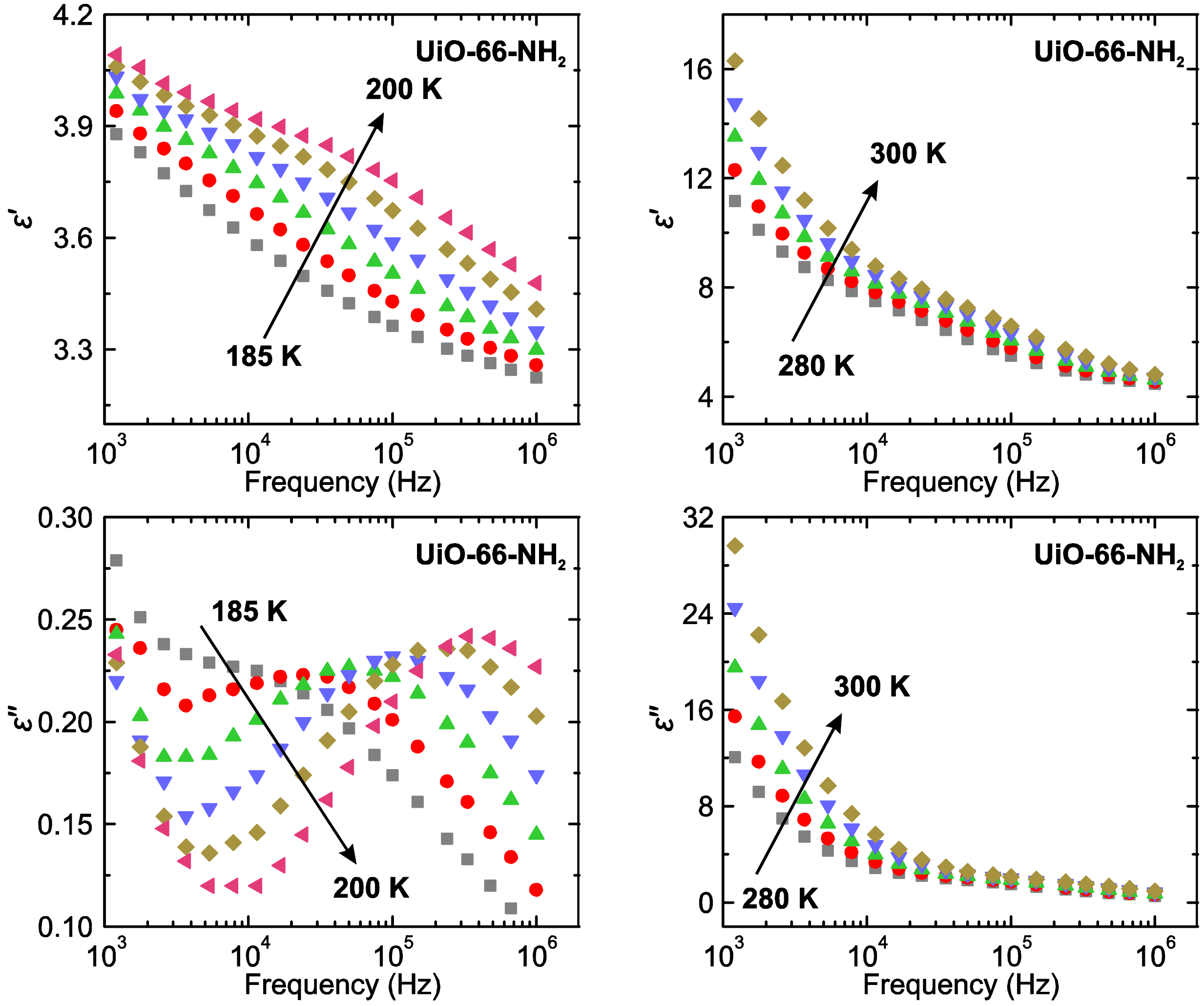

Figure S4. Frequency dependence of the complex dielectric permittivity of UiO-66-NH_2 hydrated MOF in two temperature ranges, which correspond to process P1 (left) and processes P2-P3 (right).

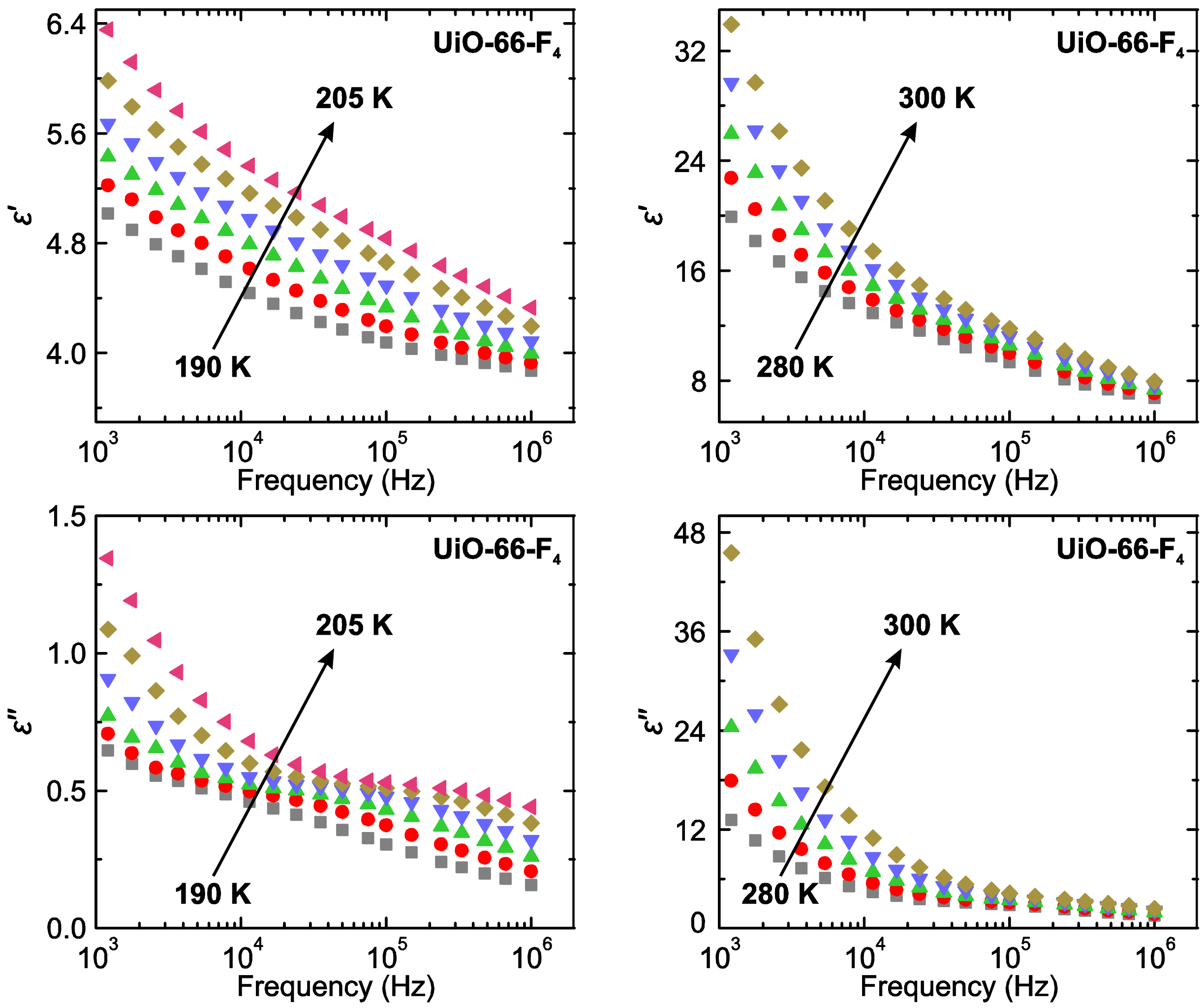

Figure S5. Frequency dependence of the complex dielectric permittivity of UiO-66-F_4 hydrated MOF in two temperature ranges, which correspond to process P1 (left) and processes P2-P3 (right).
